# Multidetector Computed Tomography Features in Differentiating Exophytic Renal Angiomyolipoma from Retroperitoneal Liposarcoma: A Strobe-Compliant Observational Study

**DOI:** 10.1097/MD.0000000000001521

**Published:** 2015-09-18

**Authors:** Qiushi Wang, Yu-Hsiang Juan, Yong Li, Jia-Jun Xie, Hui Liu, Hongfei Huang, Zaiyi Liu, Junhui Zheng, Ujwala S. Saboo, Sachin S. Saboo, Changhong Liang

**Affiliations:** From the Department of Radiology, Guangdong General Hospital, Guangdong Academy of Medical Sciences, GuangZhou, GuangDong, China (QW, J-JX, HL, HH, ZL, JZ, CL); Department of Medical Imaging and Intervention, Chang Gung Memorial Hospital, Linkou, Taoyuan, Taiwan (Y-HJ); Healthy Aging Research Center, Chang Gung University, Taoyuan, Taiwan (Y-HJ); Department of General Surgery, Guangdong General Hospital, Guangdong Academy of Medical Sciences, GuangZhou, GuangDong, China (YL); Schepens Eye Research Institute, Harvard Medical School, Boston, MA (USS); and Department of Radiology, University of Texas Southwestern Medical Center, Dallas, TX (SSS).

## Abstract

This study aims to evaluate the multidetector computed tomography (CT) imaging features in differentiating exophytic renal angiomyolipoma (AML) from retroperitoneal liposarcoma.

We retrospectively enrolled 42 patients with confirmed exophytic renal AML (31 patients) or retroperitoneal liposarcoma (11 patients) during 8 years period to assess: renal parenchymal defect at site of tumor contact, supply from branches of renal artery, tumoral vessel extending through the renal parenchyma, dilated intratumoral vessels, hemorrhage, non–fat-containing intratumoral nodules with postcontrast enhancement, calcification, renal sinus enlargement, anterior displacement of kidneys, and other associated AML.

Renal parenchymal defect, renal arterial blood supply, tumoral vessel through the renal parenchyma, dilated intratumoral vessels, intratumoral/perirenal hemorrhage, renal sinus enlargement, and associated AML were seen only or mainly in exophytic renal AML (all *P* value < 0.05); however, non–fat-attenuating enhancing intratumoral nodules, intratumoral calcification, and anterior displacement of the kidney were more common in liposarcoma (all *P* value < 0.05).

AMLs reveal renal parenchymal defect at the site of tumor contact, supply from renal artery, tumoral vessel extending through the renal parenchyma, dilated intratumoral vessels, intratumoral and/or perirenal hemorrhage, renal sinus enlargement, and associated AML. Non–fat-attenuating enhancing intratumoral nodules, intratumoral calcifications, and anterior displacement of kidney were more commonly seen in liposarcoma.

## INTRODUCTION

Renal angiomyolipoma (AML) and retroperitoneal liposarcoma are both common retroperitoneal masses containing adipose tissue. Small renal AML can be easily diagnosed on multidetector computed tomography (CT) based on the presence of fat; however, large AMLs are usually exophytic and extend into perinephric space; thus, both exophytic renal AMLs and retroperitoneal liposarcomas may appear as large fat-containing perinephric masses, rendering difficulty in differentiating these 2 entities. The same diagnostic dilemma exists not only on CT images, but even on histopathologic examinations of small samples. The evaluation of distinguishing imaging features between exophytic renal AML and retroperitoneal liposarcoma has substantial clinical impact on treatment modality because AMLs are benign tumors with good prognosis and can be treated by embolization, local resection, or surveillance, whereas retroperitoneal liposarcomas are malignant with a bleak prognosis, and thus require more aggressive therapy, including radical surgery, even irradiation.^1–3^ Although some prior studies had evaluated several CT features in distinguishing exophytic AML from retroperitoneal liposarcoma, some of the results are inconsistent; moreover, they did not explore the value of the feeding artery of the tumor and the change in renal sinus.^4–6^ Therefore, the aim of this study was to evaluate the CT imaging features in differentiating exophytic renal AML from retroperitoneal liposarcoma.

## MATERIALS AND METHODS

### Patient Selection Criteria

This retrospective study was conducted in a single tertiary care center with approval from our research ethics committee (Research Ethics Committee, Guangdong General Hospital, Guangdong Academy of Medical Sciences) and waived the need for patient informed consent. We performed a complete search of our local database from June 2006 to May 2014 for all patients with confirmed exophytic renal AML or retroperitoneal liposarcoma in the perirenal space. A patient was diagnosed as exophytic renal AML if the lesion satisfied all of the following criteria: the center of the lesion was outside the kidney, <50% of the lesion was encompassed by renal parenchyma,^4^ the maximum diameter of lesion was >5 cm, the mass contained visible fatty component on CT images, and there was histopathologic confirmation by surgery or the lesion was stable on follow-up serial CT scans for ≥12 months. Patients with liposarcomas were included if all of the following criteria were fulfilled: the mass was located in the retroperitoneum, the mass contained visible fatty component on CT images, and there was histopathologic confirmation. As a result, 42 patients were included, comprising of 31 patients with exophytic renal AMLs including 15 patients confirmed by surgery, 16 patients diagnosed by follow-up CT scans (26 female; median age: 52 years, range 14–76 years), and 11 patients with retroperitoneal liposarcomas, all confirmed by surgery (6 female; median age: 58 years, range: 39–83 years).

### CT Examinations Protocol

All patients were examined either with an 8-slice CT scanner (n = 9; GE LightSpeed Qx/I Ultra, General Electric Healthcare, Milwaukee, WI), 64-slice CT scanner (n = 15; GE Healthcare Light-Speed VCT, General Electric Healthcare) or 256-slice CT scanner (n = 18, Brilliance iCT, Philips Healthcare, Cleveland, OH) in supine position. Scanning parameters were as follows: tube voltage of 120 kVp; tube current of 200–400 mA (depending on patient size); beam collimation and rotation time, 1.28 mm and 0.35 seconds for 8-slice CT scanner, 0.625 mm and 0.35 seconds for 64-slice CT scanner, 0.75 mm and 0.27 seconds for 256-slice CT scanner respectively; reconstructed image thickness 3 mm in unenhanced, corticomedullary, and nephrographic phases, and if necessary, image thickness 1.25 mm with a interval of 1.25 mm was reconstructed for analysis. All patients underwent precontrast scan followed by corticomedullary and nephrographic phases scanning at 30 and 90 seconds delay following intravenous injection of 90 to 100 mL nonionic contrast medium (Iopamiro 370 mg I/mL, Bracco; Ultravist 370 mg I/mL, Bayer Healthcare or Omnipaque 350 mg I/mL, GE Healthcare) at a rate of 3.5 mL/s using an automatic injector (MissouriTM, Ulrich Medical, Ulm, Germany). Oblique coronal or sagittal reconstruction was performed for analysis.

### CT Image Analysis

**FIGURE 1:**
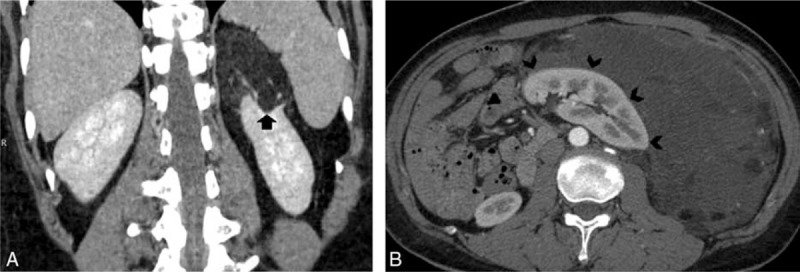
A, A 41-year-old man with exophytic renal angiomyolipoma and B, a 41-year-old female with retroperitoneal liposarcoma. Coronal contrast-enhanced multidetector computed tomography (CT), A, shows a fat-containing mass arising from the left kidney with a sharp parenchymal defect at the site of tumor contact (black arrow). Contrast-enhanced axial CT image, B, reveals a large fatty mass in the left perinephric space that partially engulfs the left kidney with mass effect displacing the abdominal contents to the midline and contralateral side. The interface between liposarcoma with kidney is smooth (black arrowheads).

Two senior abdominal radiologists (17 and 16 years of expertise, respectively) reviewed all CT images in consensus without prior knowledge of the final clinical or pathological diagnosis. The CT features were recorded for the presence or absence of the following: renal parenchymal defect at the site of tumor contact (Figure 1), tumoral vascular supply from renal artery branches, tumoral vessels extension through the renal parenchyma, dilated intratumoral vessels, intratumoral or perirenal hemorrhage, non–fat-attenuating enhancing intratumoral nodules, calcification, renal sinus enlargement, anterior displacement of the kidney, and other identifiable AML.

### Statistical Analysis

The frequency of these findings between patients with exophytic renal AMLs and retroperitoneal liposarcomas was compared using the Fisher exact test. Data analysis was performed with SPSS 13.0 software (SPSS Inc. Chicago, IL). A *P* value of < 0.05 was considered statistically significant.

## RESULTS

A summary of the CT features of exophytic renal AML and retroperitoneal liposarcoma is listed in Table 1. Renal parenchymal defect at the site of tumor contact, dilated intratumoral vessels, supply from branches of renal artery, tumoral vessels extending through the renal parenchyma, hemorrhage, associated AML, and renal sinus enlargement were seen either exclusively in or more commonly in exophytic renal AML (*P* < 0.05). On the contrary, anterior displacement of the ipsilateral kidney, intratumoral calcification, non–fat-attenuating enhancing intratumoral nodules, were primarily seen in retroperitoneal liposarcoma (*P* < 0.05).

**TABLE 1:**
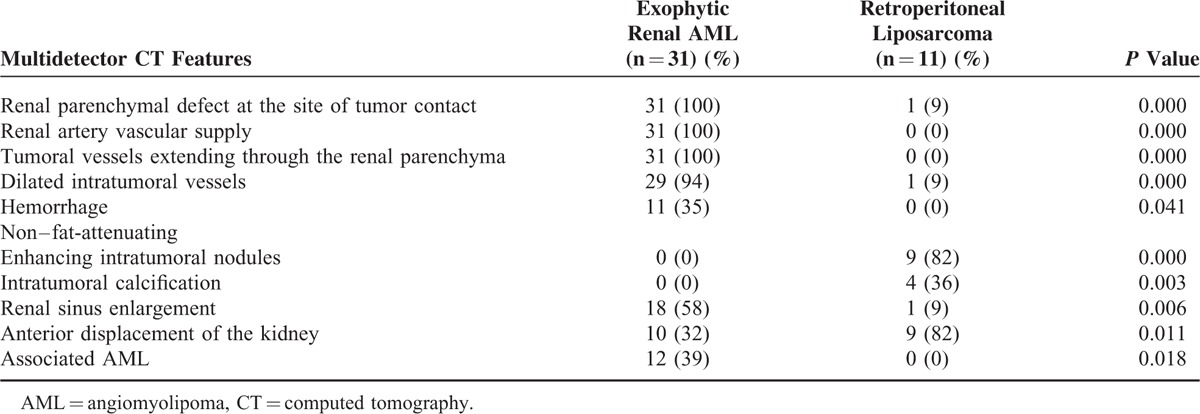
Summary of Multidetector CT Features of Differentiating Exophytic Renal AML from Retroperitoneal Liposarcoma

### Renal Parenchymal Defect at the Site of Tumor Contact

Renal parenchymal defect at the site of tumor contact was identified definitely in all patients of exophytic renal AML (31/31, 100 %) with a classic “beak sign”^6,7^ (Figure 1A). In contrast, this feature was identified in only 1 of 11 cases of retroperitoneal liposarcoma (1/11, 9%). In most of the cases, smooth interface was seen between the retroperitoneal liposarcoma and the ipsilateral kidney with intact renal parenchyma (Figure 1B). In 1 case of liposarcoma engulfing the kidney containing a renal cyst, a renal parenchymal defect was seen at the site of contact with renal cyst; however, this feature was still absent at the site of liposarcoma interface with kidney. In another case of liposarcoma, although its interface with kidney was angulated, the renal parenchyma was still intact without any defect.

### Supply from Renal Artery Branches

**FIGURE 2:**
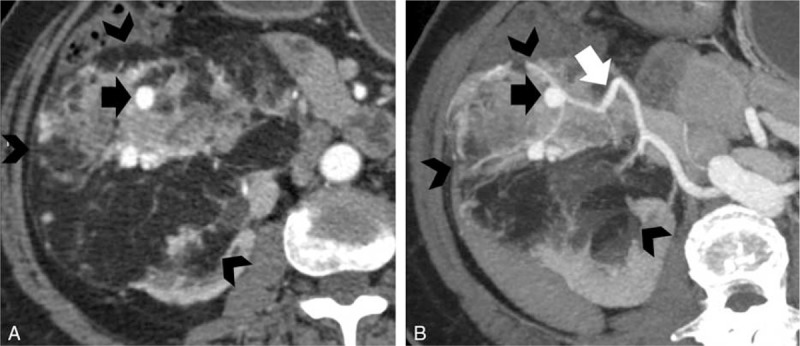
A 56-year-old man with exophytic right renal angiomyolipoma. A, Axial and B, thin maximum-intensity projection images show a fat-containing mass arising from right kidney (black arrowheads). The feeding artery of the mass is from right renal artery (white arrow), and the images also reveal aneurysm (black arrow) of the feeding artery.

**FIGURE 3:**
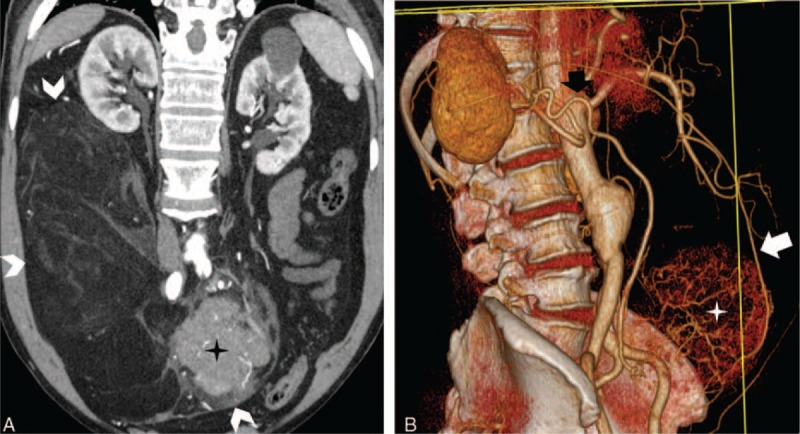
A 64-year-old man with liposarcoma. A, Oblique coronal contrast-enhanced computed tomography image reveals a large fatty tumor arising from right retroperitoneal space (white arrowheads) and a well-defined soft tissue mass within the tumor (cross). B, three-dimensional volume-rendering reconstruction image shows the supply vessel (black and white arrow) of the tumor originating from right adrenal artery and circumventing the kidney.

Feeding arteries originated from renal artery branches for all exophytic renal AMLs (31/31, 100%; Figure 2). As for retroperitoneal liposarcoma, other than 3 cases that we have failed to clearly reveal the feeding artery, all other cases (8/11, 73%) had arterial supplies other than renal arteries, including adrenal artery in 3 cases (Figure 3), and single case of right lumbar artery, inferior mesenteric artery, left intercostal artery, right external iliac artery separately, and the last case with combined left adrenal, left intercostal and left lumbar arteries.

### Tumoral Vessels Extending through the Renal Parenchyma

**FIGURE 4:**
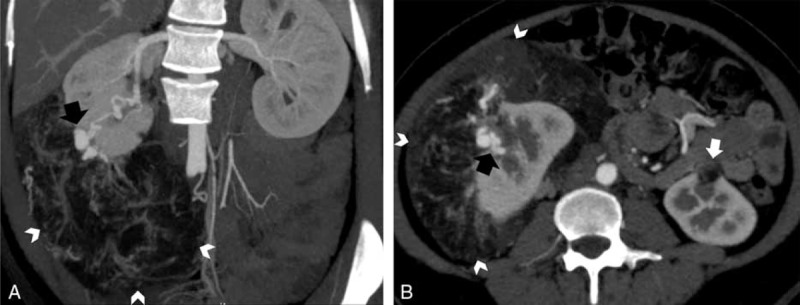
A 28-year-old female with bilateral angiomyolipoma. A, Coronal maximum-intensity projection reformation image shows a large fat-attenuation mass (white arrowheads) arising from the right kidney and a dilated tumoral vessel (black arrow) extending from the mass through the renal parenchymal defect into the renal sinus. B, Axial contrast-enhanced computed tomography also reveals a small fat-containing mass similar to the large one arising from left kidney (white arrow).

Tumoral vessels were seen extending and transversing through the renal parenchyma to connect with renal sinus vessels in all exophytic renal AMLs (31/31, 100%; Figure 4); however, tumor vessels circumvented the kidney rather than transversing through it in cases of liposarcomas (0/11, 0%; Figure 3).

### Dilated Intratumoral Vessels

Dilated intratumoral vessels were present in 29 cases of exophytic renal AML and were intensively distributed (29/31, 94 %; Figure 4). Within 11 of these cases, aneurysmal tortuous dilated vessels were observed. On the contrary, liposarcomas had relatively less vascularity, with fine and disperse vessels, and only 1 case had dilated intratumoral vessels (1/11, 9 %).

### Hemorrhage

Combined intratumoral and/or perirenal hemorrhage were present only in 11 AMLs (11/31, 35%). Among them, 5 cases manifested intratumoral hemorrhage, 1 case exhibited perirenal hemorrhage, and 5 cases demonstrated intratumoral and perirenal hemorrhage simultaneously. Among these cases, 10 presented with dilated vessels within tumors. On the contrary, none of the case of liposarcoma (0/11, 0%) was associated with hemorrhage.

### Non–Fat-Containing Intratumoral Nodules with Postcontrast Enhancement

**FIGURE 5:**
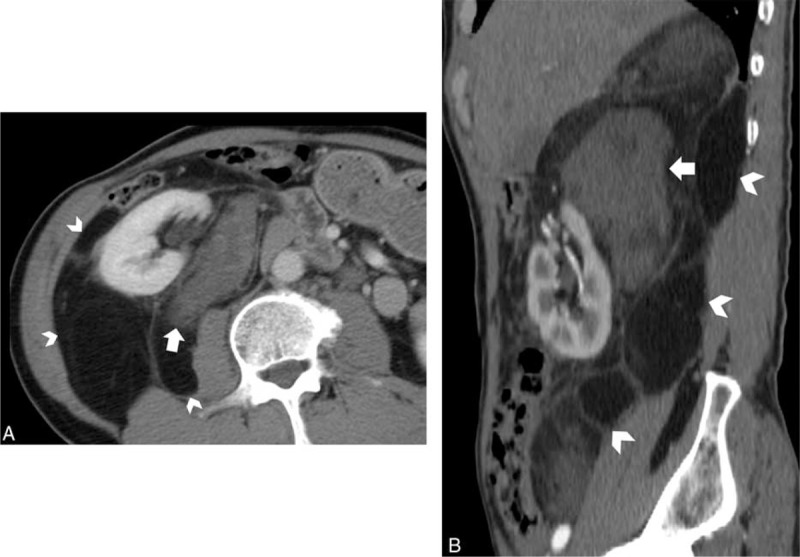
A 57-year-old man with retroperitoneal liposarcoma. A, Axial and B, oblique sagittal contrast-enhanced computed tomography images reveal a large fat-containing mass engulfing the right kidney (white arrowheads) and also a well-defined soft tissue intratumoral nodule with mild enhancement within the tumor (white arrow).

Non–fat-containing intratumoral nodule with postcontrast enhancement was not seen in any of the exophytic renal AMLs (0/31, 0%). The non–fat-attenuating areas with postcontrast enhancement were patchy and not nodular for all exophytic renal AMLs, except for 1 case in which the hemorrhagic focus appeared as isodense nonenhancing intratumoral nodule compared with normal kidney. On the contrary, in 9 patients with liposarcomas (9/11, 82%), the non–fat-attenuating areas appeared as enhancing intratumoral nodules, with the solid areas being embedded within the fat (Figure 5).

### Calcification

**FIGURE 6:**
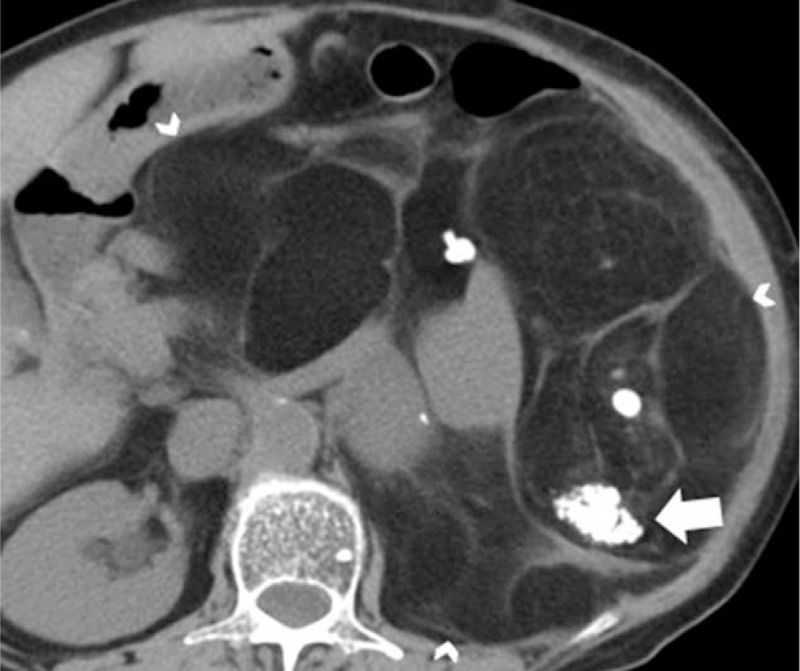
A 67-year-old woman with a large left retroperitoneal liposarcoma. Non–contrast-enhanced computed tomography scan shows a large fat-containing mass (white arrowheads) with coarse calcification (white arrow).

Coarse, punctuate, or granular calcifications were seen in 4 cases of 11 liposarcomas (4/11, 36%; Figure 6) but not in AMLs (0/31, 0%).

### Renal Sinus Enlargement

**FIGURE 7:**
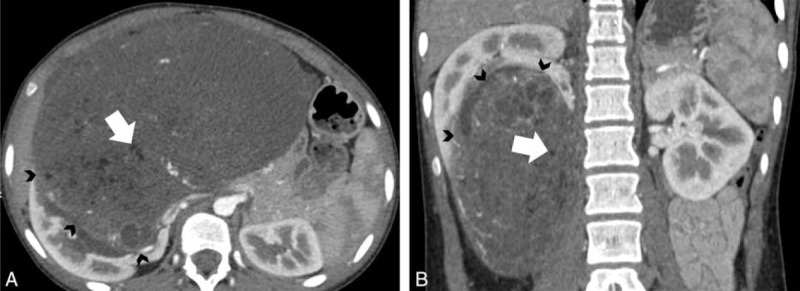
A 14-year-old woman with angiomyolipoma. A, Axial and B, coronal contrast-enhanced computed tomography images show fatty (white arrow) mass arising from right kidney with enlargement of renal sinus (black arrowheads).

Renal sinus enlargement was seen in 18 of 31 cases of AML (18/31, 58%; Figure 7); however, only in 1 case of liposarcoma (1/11, 9%).

### Anterior Displacement of the Kidney

Anterior displacement of ipsilateral kidney was seen in 10 cases (10/31, 32%) of exophytic AMLs. Among these, 9 cases had tumor origin from posterior renal parenchyma and the remaining case was associated with perirenal hemorrhage. It is important to note that the posterior border of the displaced kidney did not exceed the anterior border of adjacent vertebra in these cases of AMLs, and neither did the kidney rotate. On the contrary, 9 cases of liposarcomas growing around the kidney caused severe anterior ipsilateral renal displacement (9/11, 82%). In 7 of these 9 cases, the posterior border of the anteriorly displaced kidney exceeded the anterior border of adjacent vertebrae (Figure 1B), and in 4 of them, the displaced kidney had obvious rotation.

### Other Associated AML

Additional AMLs were present either in the ipsilateral or contralateral kidney in 12 cases of AML (12/31, 39%; Figure 4), of which 4 cases had tuberous sclerosis complex. Associated AMLs were seen in none of the patient with liposarcoma (0/11, 0%).

## DISCUSSION

Establishing confident diagnostic CT features for differentiating exophytic renal AML with retroperitoneal liposarcoma has significant clinical impact in making clinical decision. Exophytic renal AML is a benign renal neoplasm, although a small subset of patients may present with life-threatening hemorrhage, hence if the diagnosis is confirmed before treatment, clinical and imaging follow-ups are sufficient for most cases. As for the patients with recurrent episodes of hemorrhage or massive bleeding, the tumor may be treated by embolization or partial nephrectomy.^1,8^ Moreover, recurrence after surgery is extremely rare in patients with AML.^2,9,10^ On the contrary, retroperitoneal liposarcoma is the most common primary retroperitoneal malignancy and is associated with unfavorable prognosis. Surgical resection is a main treatment for primary liposarcoma along with other aggressive local therapy, including irradiation. The overall 5-year survival rate of patients with retroperitoneal liposarcoma is about 23% to 46%.^3^

Accurate imaging differentiation between AML and liposarcoma is important for the prognosis and treatment because histologic examination may be inconclusive when the smooth muscle cells are scant and atypical.^11^ The CT features can help to resolve the existing dilemma between exophytic renal AML and retroperitoneal liposarcoma.

Renal parenchymal defect at the site of tumor contact has been reported as a reliable indicator in identifying exophytic renal AML.^4–6^ The integrity of renal contour and renal parenchyma is the most important CT sign for differentiating renal from nonrenal origin perirenal space tumors.^7^ As AML arises from the renal parenchyma, a parenchymal defect would be expected at its origin. Our study findings of renal parenchyma defect in all the cases of exophytic renal AMLs correlate with previous literatures.^4–6^ As the liposarcoma arises from the retroperitoneal adipose tissue, the interface between liposarcoma and kidney would be expected to be intact. In our present study, only 1 case of liposarcoma with solid tumoral tissue directly invading the ipsilateral kidney showed renal parenchymal defect at the site of tumor contact.

Dilated intratumoral vessels and the presence of renal parenchymal vascular pedicle are 2 helpful signs in differentiating these 2 tumor entities.^4,5^ Israel et al^5^ showed that the presence of enlarged intratumoral vessels is a strong indicator for an AML, whereas Ellingson et al^4^ emphasized the importance of renal parenchymal vascular pedicle. In the present study, in addition to the 2 signs mentioned above, we also analyzed the feeding artery of these 2 tumors. Our results revealed that all cases of exophytic renal AML had supply from branches of renal artery and intratumoral vessels extending through the renal parenchyma. AMLs are vascular tumors arising from renal parenchyma; hence, they would be expected to share similar vasculature with the renal parenchyma. Since liposarcomas arise from the adjacent retroperitoneal adipose tissue, it is reasonable to expect blood supply from other retroperitoneal vessels.

The difference in distribution and size of vessels can also be accounted to their histology. As AML is composed of mature adipose tissue, spindle-shaped or/and epithelioid smooth muscle cells and abnormal thick-walled blood vessels, it commonly reveals dilated intratumoral vessels on contrast-enhanced CT.^12^ In the present study, dilated intratumoral vessels were present in 29 cases (94%) of exophytic renal AML, of which, 11 cases exhibited aneurysmal dilatation of vessels; however, liposarcomas are relatively avascular, especially in well-differentiated subtype of liposarcomas. Our study showed that 10 of 11 cases of liposarcomas show only thin sparse blood vessels, as opposed to the dilated vessels in AML.

The blood flow to AML increases with AML size growth, thereby causing vessel dilatation and thickening. The profuse and abnormal elastin-poor vascular structures in the tumor permit aneurysm formation and intratumoral or perirenal hemorrhage.^12,13^ In contrast, in general, liposarcomas are relatively avascular lesions, whereas hemorrhage is scarce. In the present study, 11 cases (35%) of exophytic renal AML reveal signs of hemorrhage, but none of liposarcomas were hemorrhagic. The presence of hemorrhage is a good indicator to differentiate these 2 tumor entities.

Our study showed that 9 of 11 liposarcomas (82%) exhibit non–fat-attenuating enhancing intratumoral soft tissue nodule, which is in concordance to previous observations.^6^ The soft tissue nodules within the liposarcoma may represent dedifferentiated or myxoid components. Although these are embedded within the tumor, they are clearly demarcated from the surrounding fatty components on both CT and histopathology.^11,14^ Occasionally, exophytic renal AML can have non–fat-attenuating enhancing nodules within the tumor,^6^ which represent a combination of smooth muscle, mature adipose soft tissue and abnormal vessels, but usually with an ill-defined demarcation from fatty components, as presented in our cases.

The previous data showed that calcification within the tumor might help to differentiate between exophytic AML and retroperitoneal liposarcoma.^4^ Calcifications within exophytic renal AML are only occasionally detected.^15^ On the contrary, calcification is easily seen in liposarcoma and has been considered a feature of poor prognosis, often indicating dedifferentiation.^16,17^ The results of our study demonstrated that 4 of 11 liposarcomas cases (36%) presented with intratumoral calcification, whereas calcification was seen in none of AML cases.

The renal sinus is a central spacious cavity formed by the extension of the perinephric space into the deep recess located at the medial border of the kidney. The renal sinus is surrounded by the kidney parenchyma laterally; therefore, the renal sinus can be secondarily involved by the surrounding renal parenchymal and adjacent retroperitoneal lesions. There are few studies illustrating the cross-sectional imaging appearance of AML or liposarcoma within the renal sinus, but the associations between tumor entities and renal sinus enlargement were not studied.^18–20^ In our present study, 18 of 31 AML cases (58%) originated from the adjacent renal parenchyma close to renal sinus fat and extended into the renal sinus, thus causing its enlargement. Only 1 of 11 cases of liposarcoma (9%) exhibited renal sinus enlargement along with encompassment of the renal artery and renal vein.

Our study revealed that retroperitoneal liposarcoma exhibited more noticeable anterior displacement of the kidney than exophytic renal AML. This finding can be secondary to the slow and progressive anterior displacement by retroperitoneal liposarcoma, and therefore, the symptoms from renal capsular traction are delayed and less pronounced as compared with the direct capsular distension exhibited by exophytic AML. Wu et al^7^ suggested that the displacement of kidney with accompanying rotation of renal axis was more commonly seen in nonrenal tumors than renal tumors, which the results are further confirmed by the results of our study.

Israel et al^5^ showed that the presence of other associated AMLs in the ipsilateral or contralateral kidney, independent of the dominate lesion, was a strong indicator of AML, although their study did not include the cases of tuberous sclerosis. As for our study, 4 cases of tuberous sclerosis were not excluded, because these cases manifested with abdominal abnormalities prior to the confirmed diagnosis of tuberous sclerosis. In the present study, associated AMLs were seen in 12 cases of AML but in none cases of liposarcoma and can thus facilitate to reliably differentiated exophytic renal AML from retroperitoneal liposarcoma.

Our study had 2 limitations. The 42 cases were all retrospectively searched from the database from our single tertiary center. Further studies with multicenter results may further confirm our findings. In addition, histopathologic proof of diagnosis was not available in some exophytic renal AMLs, but the diagnosis was further supported in these cases as the lesions remain stationary without treatment for at least 12 months.

In the evaluation of a large fat-containing perinephric mass on CT, the presence of renal parenchymal defect at the site of tumor contact, supply from renal artery, tumoral vessel extending through the renal parenchyma, dilated intratumoral vessels, intratumoral and/or perirenal hemorrhage, renal sinus enlargement, and associated AML were either only seen in or significantly more common in exophytic renal AML; whereas non–fat-attenuating enhancing intratumoral soft tissue nodules, intratumoral calcifications, and anterior displacement of kidney were more commonly seen in retroperitoneal liposarcoma.
